# Systematic Analysis of NRAMP Family Genes in *Areca catechu* and Its Response to Zn/Fe Deficiency Stress

**DOI:** 10.3390/ijms24087383

**Published:** 2023-04-17

**Authors:** Guangzhen Zhou, Qiyuan An, Zheng Liu, Yinglang Wan, Wenlong Bao

**Affiliations:** 1 Hainan Key Laboratory for Sustainable Utilization of Tropical Bioresources, College of Tropical Crops, Hainan University, Haikou 570228, Chinaylwan@hainanu.edu.cn (Y.W.); 2 Key Laboratory for Quality Regulation of Tropical Horticultural Crops of Hainan Province, School of Horticulture, Hainan University, Haikou 570228, China; 3 Hainan Yazhou Bay Seed Laboratory, Sanya Nanfan Research Institute, Hainan University, Sanya 572025, China

**Keywords:** *Areca catechu*, NRAMP, Fe and Zn deficiency, stress response, gene expression

## Abstract

*Areca catechu* is a commercially important medicinal plant widely cultivated in tropical regions. The natural resistance-associated macrophage protein (NRAMP) is widespread in plants and plays critical roles in transporting metal ions, plant growth, and development. However, the information on *NRAMPs* in *A. catechu* is quite limited. In this study, we identified 12 *NRAMPs* genes in the areca genome, which were classified into five groups by phylogenetic analysis. Subcellular localization analysis reveals that, except for NRAMP2, NRAMP3, and NRAMP11, which are localized in chloroplasts, all other NRAMPs are localized on the plasma membrane. Genomic distribution analysis shows that 12 *NRAMPs* genes are unevenly spread on seven chromosomes. Sequence analysis shows that motif 1 and motif 6 are highly conserved motifs in 12 *NRAMPs*. Synteny analysis provided deep insight into the evolutionary characteristics of *AcNRAMP* genes. Among the *A. catechu* and the other three representative species, we identified a total of 19 syntenic gene pairs. Analysis of Ka/Ks values indicates that *AcNRAMP* genes are subjected to purifying selection in the evolutionary process. Analysis of cis-acting elements reveals that *AcNRAMP* genes promoter sequences contain light-responsive elements, defense- and stress-responsive elements, and plant growth/development-responsive elements. Expression profiling confirms distinct expression patterns of *AcNRAMP* genes in different organs and responses to Zn/Fe deficiency stress in leaves and roots. Taken together, our results lay a foundation for further exploration of the *AcNRAMPs* regulatory function in areca response to Fe and Zn deficiency.

## 1. Introduction

Metal ions play a key role in maintaining the stability of plant physiological and biochemical functions. For example, Fe is an essential element involved in cell respiration, photosynthesis, and the catalytic reaction of metalloproteins, while Zn is a structural cofactor of a variety of enzymes and proteins [1,2]. The balance of metal ions in the plant is also necessary for plant development. A high concentration of free iron (Fe^3+^/Fe^2+^) generates oxygen and hydroxyl radicals through the Fenton reaction, which causes a redox reaction and accumulation of superoxide compounds, resulting in intracellular damage to DNA and lipids [3]. Meanwhile, Fe deficiency can also affect plant growth and development. Similarly, excess zinc binds to sulfur, nitrogen, and oxygen-containing functional groups in biological molecules due to its upregulated high affinity, which, in turn, interferes with their biological activities. Zn deficiency leads to the oxidative destruction of chlorophyll, lipids, and proteins [4].

The maintenance of metal ion balance in plants is mainly achieved through the synergies of various metal transporters, including zinc-regulated transporters iron-regulated transporter-like proteins (ZIP), NRAMP, cation diffusion facilitator proteins (CDF), heavy metal ATPase (HMA), yellow stripe-like (YSL), and ATP-binding cassette (ABC) transporters [5,6]. Of these, the NRAMP proteins are a vital membrane transporter family that exist widely in plants, which are mainly involved in the transport of divalent metal cations such as Zn, Fe, and Cu. Moreover, the NRAMP proteins play a principal role in regulating and maintaining the homeostasis of Mn/Fe in plants [7,8]. NRAMP was first found in mice. Subsequently, NRAMP was verified as a metal transporter in diverse organisms including fungi, animals, and plants [9,10]. In the previous study, the roles of NRAMP proteins have been explored in some plant species, including model plants, forest trees, and horticultural plants [10,11,12,13,14,15]. Different NRAMPs enable the trafficking of different essential or toxic metal ions. For instance, *Arabidopsis* AtNRAMP6 is considered a transporter of cadmium [16], while AtNRAMP1 is a Mn transporter. AtNRAMP3 and AtNRAMP4 are responsible for the transportation of Fe and Mn [17]. Rice OsNRAMP3 is identified as a Mn transporter, while OsNRAMP5 is identified as a Mn/Cd transporter [18,19]. In addition, OsNRAMP4 plays a key role in the trafficking of trivalent Al ions [20]. In summary, NRAMP proteins are critical members of the membrane transporter family involved in metal ion transport, absorption, intracellular transport, and detoxification.

*Areca catechu* (areca palm) is an evergreen tree of the genus Areca in the palm family, which is native to Malaysia and widely cultivated in tropical Asia. In our previous studies, we treated areca seedlings with Fe and Zn deficiency and found a significant decrease in iron and zinc content in the roots [21]. In this study, 12 *NRAMP* genes were identified from the whole genome of *A. catechu*, and their sequence characteristics, gene structure, phylogeny, promoter sequence, and collinearity were analyzed. Furthermore, the expression levels of these genes in different tissues and under the condition of Fe and Zn deficiency were studied. This work provides a basis for further investigating *NRAMP* function and areca response to Fe and Zn deficiency.

## 2. Results

### 2.1. Identification and Characterization of AcNRAMPs

**Figure 1:**
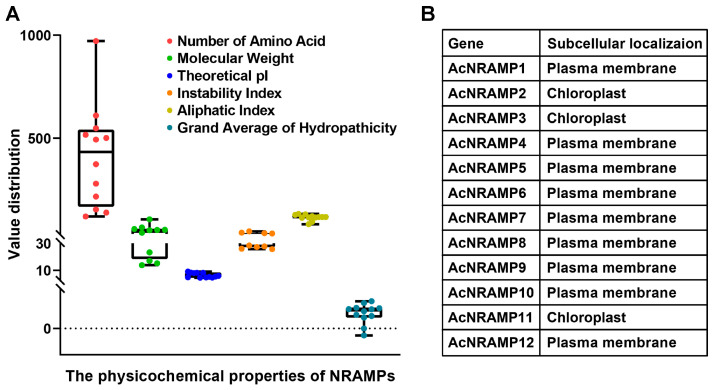
Physicochemical properties of AcNRAMPs (**A**) and prediction of subcellular localization (**B**).

Twelve *AcNRAMP* candidates were identified in the *A. catechu* genome (Figure 1A, Table S2). The amino acid lengths of 12 AcNRAMP proteins altered from 120 (AcNRAMP5) to 971 (AcNRAMP2), while the molecular weight changed from 13.86–106.01 KDa. The theoretical isoelectric point (pI) of AcNRAMP proteins varied from 4.5 (AcNRAMP10)–9.14 (AcNRAMP5). The instability index (Ii) ranged from 25.75 (AcNRAMP10) to 47.77 (AcNRAMP2). Only two AcNRAMP proteins had Ii larger than 40. The aliphatic index (Ai) analysis shows that AcNRAMP2 has the maximum Ai at 82.84, while AcNRAMP12 has the minimum Ai at 132.44. In addition, the grand average of hydropathicity (GRAVY) of all AcNRAMP proteins was positive except for AcNRAMP2. Prediction of subcellular localization reveals that all AcNRAMP proteins are localized to the plasma membrane except for AcNRAMP2, AcNRAMP3, and AcNRAMP11 (Figure 1B).

### 2.2. Duplication Analysis and Phylogenetic Analysis of AcNRAMPs

**Figure 2:**
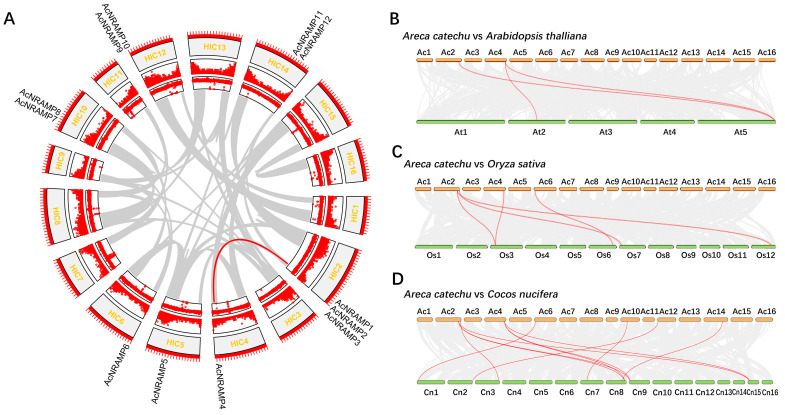
Chromosomal location of *AcNRAMP* genes. (**A**) Chromosomal location of *AcNRAMP*s and their collinear gene pairs in *A. catechu* genome. The outer ring to the inner ring represents 16 chromosomes, gene density, and GC contents, respectively. (**B**–**D**) Syntenic analysis of *AcNRAMP*s between *A. catechu* and *A. thaliana*, *A. catechu* and *O. sativa*, and *A. catechu* and *C. nucifera*, respectively. The gray lines among each chromosome represent all gene pairs between *A. catechu* and other plant species, while the red lines represent the gene pairs associated with *AcNRAMP* genes.

To understand the genomic distribution of 12 *AcNRAMP*s, their chromosomal location was analyzed. The twelve genes were located on seven chromosomes, of which chromosome two had a maximum of three genes (Figure 2A). To reveal the *AcNRAMP* collinear gene pairs, the syntenic gene analysis was carried out. The results show that only one collinear *AcNRAMP* (*AcNRAMP3*–*AcNRAMP4*) is identified in the areca genome (Figure 2A). To shed light on the possible evolutionary process of *AcNRAMP*s, the syntenic relationships of *AcNRAMP* genes with the other plant species were investigated, including dicot *A. thaliana* (belongs to the *Brassicaceae*), monocot *O. sativa* (belongs to the *Poaceae*), and *C. nucifera* (belongs to the *Palmae*). Three *AcNRAMP* homologous genes pairs are detected between *A. catechu* and *A. thaliana* (*AcNRAMP1*–*AT5G67330.1*; *AcNRAMP4*–*AT2G23150.1*; *AcNRAMP4*–*AT5G67330.1*) (Figure 2B, Table S3). Five *AcNRAMP* homologous gene pairs are identified between *A. catechu* and *O. sativa* (*AcNRAMP1*–*Os12t0581600-01*; *AcNRAMP1*–*Os03t0208500-01*; *AcNRAMP2*–*Os07t0155600-01*; *AcNRAMP4*–*Os03t0208500-01*; *AcNRAMP6*–*Os06t0676000-01*) (Figure 2C, Table S3). A total of 11 *AcNRAMP* homologous gene pairs are identified between *A. catechu* and *C. nucifera* (*AcNRAMP1*–*GZ08G0188430.1*; *AcNRAMP1*–*GZ09G0194900.1*; *AcNRAMP1*–*GZ15G0285060.1*; *AcNRAMP2*–*GZ03G0077500.1*; *AcNRAMP4*–*GZ08G0188430.1*; *AcNRAMP4*–*GZ09G0194900.1*; *AcNRAMP4*–*GZ15G0285060.1*; *AcNRAMP6*–*GZ01G0002400.1*; *AcNRAMP7*–*GZ07G0166110.1*; *AcNRAMP12*–*GZ02G0049210.1*; *AcNRAMP14*–*GZ08G0185160.1*) (Figure 2D, Table S3).

**Figure 3:**
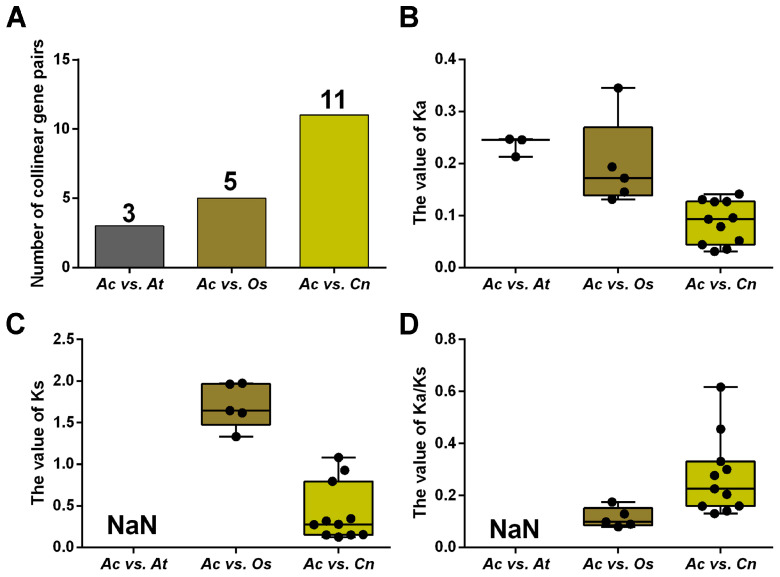
Comparison of *NRAMP* homologous genes substitution rate. (**A**) The number distribution of the collinear gene pairs in three species. Ac: *A. catechu*; At: *A. thaliana*; Os: *O. sativa*; Cn: *C. nucifera*. (**B**–**D**) The Ka, Ks, and Ka/Ks distribution of the *NRAMP* duplicated genes.

To uncover the selection pressure on *NRAMP* genes during evolution, we calculated the non-synonymous substitution rate (Ka), the synonymous substitution rate (Ks), and their ratio (Ka/Ks) of homologous *NRAMP* genes in *A. catechu*, *A. thaliana*, *O. sativa*, and *C. nucifera* (Figure 3; Table S4). The results show that the Ka/Ks values vary greatly (from 0.0797 to 0.6172) but are less than 1, suggesting purifying selection on these genes. Generally, the Ka/Ks ratios of *AcNRAMPs* and *OsNRAMPs* gene pairs are larger than those of *AcNRAMPs* and *CnNRAMPs* gene pairs, implying faster evolutionary rates of *AcNRAMP* genes on *O. sativa* than on *C. nucifera* (Table S4).

**Figure 4:**
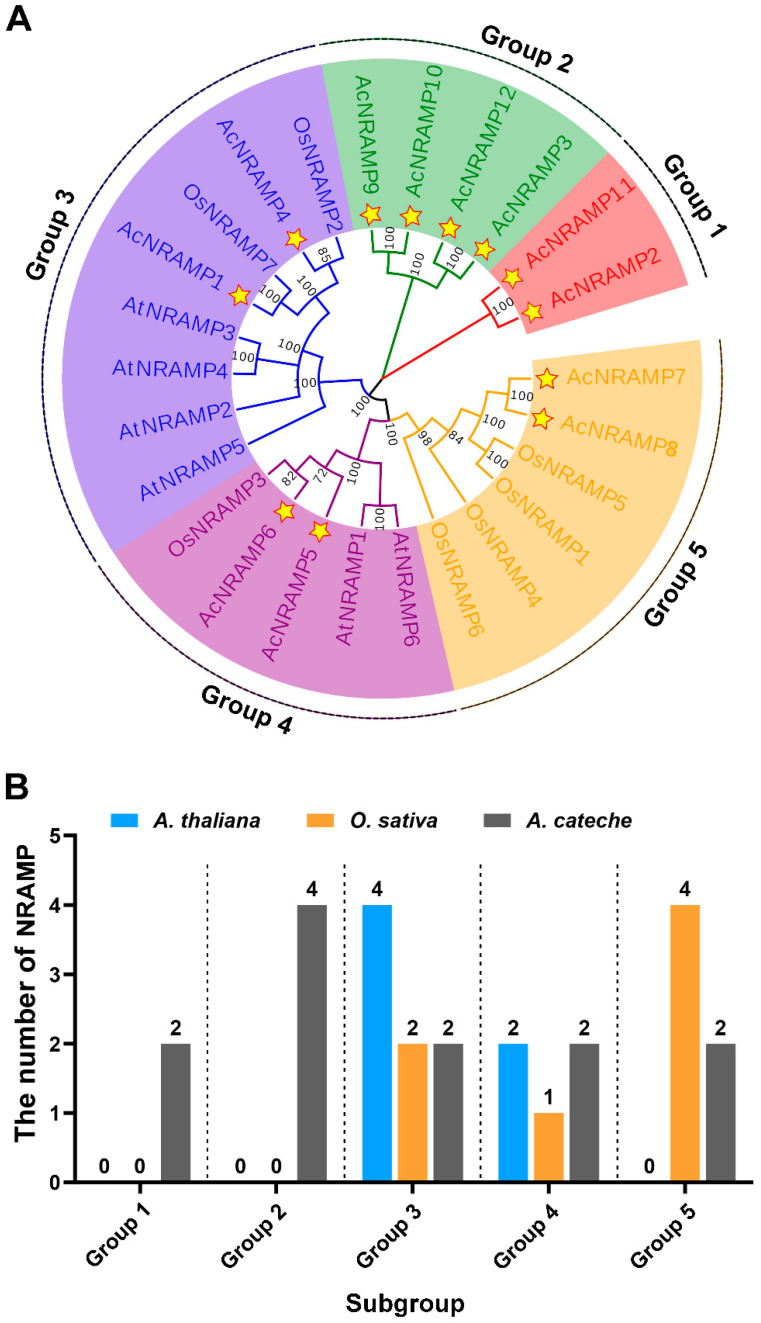
Phylogenetic relationships of NRAMP proteins in *A. catechu*, *O. sativa*, and *A. thaliana*. (**A**) The phylogenetic tree has 25 NRAMP proteins, including 12 AcNRAMP, 7 OsNRAMP, and 6 AtNRAMP). Star-marked 12 NRAMP genes of *A. catechu*. (**B**) The number of AcNRAMPs, OsNRAMP, and AtNRAMP in the different subgroups.

To clarify the phylogenetic relationships among *NRAMP* homologs in different plant species, a total of 25 *NRAMP* proteins from *A. thaliana* (6), *O. sativa* (7), and *A. catechu* (12) were used to establish a phylogenetic tree (Figure 4). According to the topology of the phylogenetic tree, 25 NRAMP were subdivided into five subgroups (group 1–group 5). *AcNRAMP* genes are distributed in all five subgroups, among which group 2 has the largest number (Figure 4).

### 2.3. Sequence Features of AcNRAMPs

**Figure 5:**
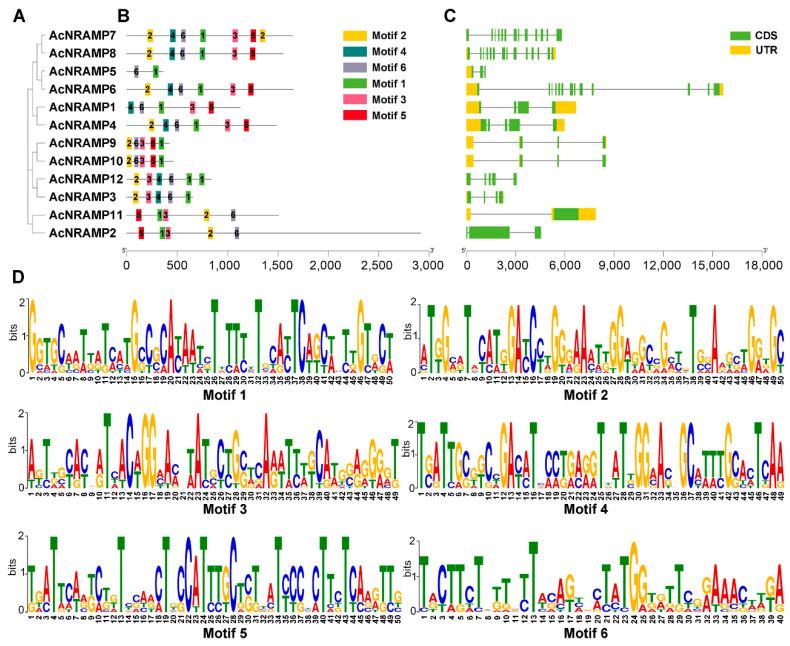
The sequence features of the *AcNRAMP* genes. (**A**) The phylogenetic tree has 12 AcNRAMPs. (**B**) The motif distribution in the 12 AcNRAMPs. The various colored rectangles represent motifs 1 to 6; the black solid lines indicate the sequences outside motifs; the bar scale below represents the number of amino acids. (**C**) The gene structures of the 12 *AcNRAMP*s. The green ellipses and orange ellipses represent exons and untranslated regions (UTR), respectively; the black solid lines linked with the ellipse indicate the introns; the bar scale below represents the gene length. (**D**) Sequence logos of conserved amino acid residues sequences. The X-axis represents the position of the different amino acids in each motif, while the Y-axis represents the bit value of each amino acid.

To illustrate the sequence features of the *AcNRAMPs*, the MEME program was used to analyze their conserved motifs. In total, six conserved motifs (motif 1–6) are found in 12 AcNRAMP protein sequences. AcNRAMP proteins within the identical subgroup have similar motifs (Figure 5A,B). Motif 1 and motif 6 are present in all proteins. Most of the AcNRAMP proteins have motifs 2 and 3. Only AcNRAMP5 proteins contain two motifs, including motif 1 and motif 6. The analysis of gene structures reveals a great change in introns number within 12 *AcNRAMP* genes (from 3 to 14), while the number distribution of UTR varies from 1 to 3 (Figure 5C). Furthermore, the frequencies of amino acids on the respective position within the sequences of six motifs in the AcNRAMPs are highly different, which are worthy of further elucidation (Figure 5D).

### 2.4. Cis-Acting Elements of AcNRAMPs

**Figure 6:**
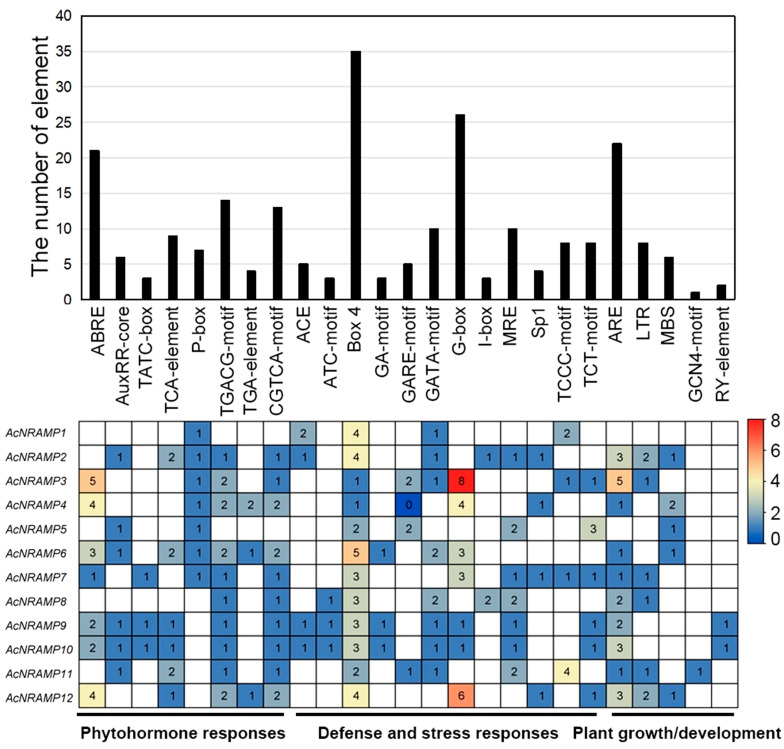
Cis-acting elements of *AcNRAMPs*. The bar chart represents the total number of cis-acting elements within each AcNRAMP. The heatmap shows the number distribution of different cis-acting elements within each *AcNRAMP*.

To gain more insight into the possible regulatory factors of 12 *AcNRAMP* genes, the Plant CARE online program was employed to analyze their cis-acting elements. We identified 25 types of cis-acting elements and classified them into three categories, namely, phytohormone-responsive elements (PREs), defense- and stress-responsive elements (DSREs), and plant growth/development-responsive elements (GDREs) (Figure 6). Of these, the PREs are the most abundant with eight types, containing the ABA-responsive elements such as ABRE, the MeJA-responsive elements such as CGTCA motif and TGACG motif, the auxin-responsive elements such as AuxRR-core, the gibberellin-responsive elements such as P-box and TATC-box, and the other elements such as TCA element and TGA element. The DSREs have 12 types, containing ATC motif, Box 4, GA motif, GARE motif, GATA motif, G-box, I-box, MRE, Sp1, TCCC motif, and TCT motif. However, only five types of GDREs are identified, containing ARE, LTR, MBS, GCN4 motif, and RY element. Moreover, *AcNRAMP9* and *AcNRAMP10* have almost identical cis-acting elements. Box 4 is present in all *AcNRAMPs* as the light-responsive element (Figure 6).

### 2.5. Expression Analysis of AcNRAMPs in Different Tissues and under Fe and Zn Deficiency

**Figure 7:**
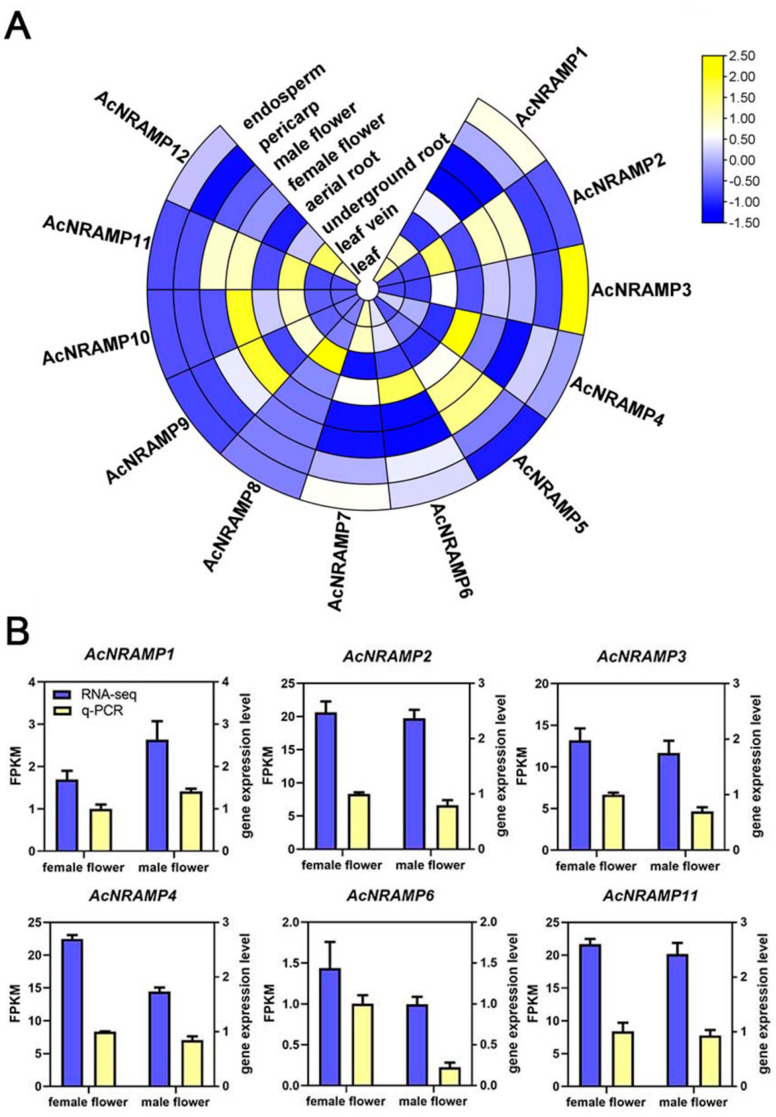
The expression patterns of 12 *AcNRAMP* genes of *A. catechu* in different tissue (**A**) and qRT-PCR validation of RNA-seq (**B**).

To elucidate the role of AcNRAMP genes in different tissue and their response to Fe and Zn deficiency, the expression profiles of *AcNRAMP* genes were analyzed by calculating gene FPKM values (Table S5). Tissue-specific expression analysis reveals that *AcNRAMP2*, *AcNRAMP8,* and *AcNRAMP11* are highly expressed in underground roots, and *AcNRAMP4* and *AcNRAMP6* are highly expressed in aerial roots. The expression levels of *AcNRAMP3* are higher in the endosperm than that in the other tissues. *AcNRAMP4* is generally highly expressed in most tissues of areca, especially in aerial roots. *AcNRAMP5* is highly expressed in flowers and pericarp. *AcNRAMP7* is absent from flowers, while the high expression level of *AcNRAMP7* is found in leaves and veins. *AcNRAMP8* is specifically up-regulated in underground roots. *AcNRAMP9* and *AcNRAMP10* are up-regulated in female flowers compared to other tissues. Furthermore, *AcNRAMP11* is only up-regulated in flowers and underground roots, while *AcNRAMP12* shows a higher expression level in leaves and veins (Figure 7A, Table S5). These results suggest that AcNRAMP proteins play vital roles in metal transportation in different areca tissues. Furthermore, the relative expression of six randomly selected *AcNRAMP* genes in female and male flower samples was detected by qRT-PCR. The results show that the gene expression trends determined by qRT-PCR and RNA-seq are highly consistent, which indicates that transcriptome data are reliable and reproducible (Figure 7B).

**Figure 8:**
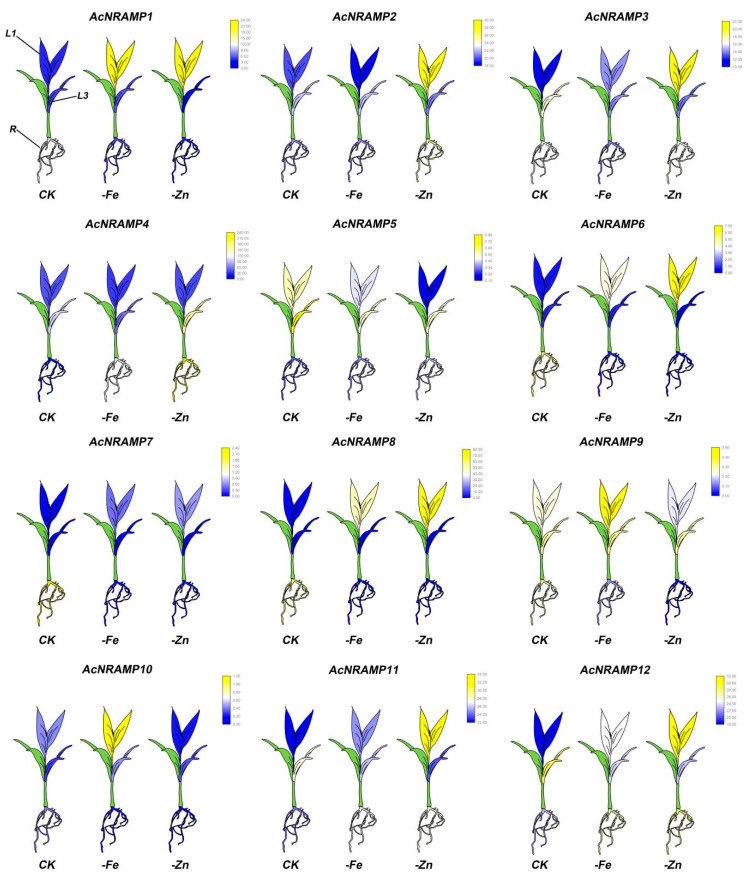
The expression patterns of 12 *AcNRAMP* genes of *A. catechu* in Fe and Zn deficiency. FPKM values were obtained by RNA-seq. Each gene has three cartoon heatmaps that represent areca seedlings in normal, Fe-deficient, and Zn-deficient conditions. The sampling mainly targeted the first leaf (L1), third leaf (L3), and root (R) of areca seedlings.

To further explore the response of *AcNRAMPs* to Fe and Zn, the expression of *AcNRAMP* in leaves and roots of areca seedlings under Fe and Zn deficiency conditions was analyzed (Table S6). In roots, *AcNRAMP1* and *AcNRAMP4* are up-regulated under Zn/Fe deficiency stress, while *AcNRAMP7* is down-regulated. *AcNRAMP3* is up-regulated in Fe-deficient roots, while *AcNRAMP10* is up-regulated in Zn-deficient roots (Figure 8). In leaves, we find that *AcNRAMP1*, *AcNRAMP9*, and *AcNRAMP10* are highly expressed in the first leaf (L1) treated with iron deficiency. Under the condition of zinc deficiency, *AcNRAMP1*, *AcNRAMP2*, *AcNRAMP3*, *AcNRAMP6*, *AcNRAMP8*, *AcNRAMP11,* and *AcNRAMP12* also show a high expression pattern in L1 (Figure 8). In addition, compared with the third leaf (L3), Zn/Fe deficiency treatment greatly affects the expression level of the *AcNRAMPs* gene in L1 (Figure 8). Furthermore, our previous study also suggests that the transcriptome data are reliable and reproducible [21]. These results suggest that the *AcNRAMPs* family plays an important role in coping with Zn/Fe deficiency stress in Areca.

## 3. Discussion

The plant *NRAMP* gene family belongs to the conserved metal transport family in natural evolution, which is mainly responsible for the absorption, transport, and intracellular stability of Fe, Mn, and other metal ions [7,22]. In addition, the *NRAMP* gene family also plays a critical regulatory role in photosynthesis, protein activity maintenance, and abiotic stress response [23]. In previous studies, the members of the *NRAMP* gene family have been identified in diverse plant genomes, including *A. thaliana* [11], *O. sativa* [12], *P. alba* [10], *P. vulgaris* [13], *T. cacao* [14], and *B. napus* [15]. In this study, we identified 12 *NRAMP* genes in the *A. catechu* genome. Notably, the *NRAMP* genes in *Arabidopsis* were classified into two subfamilies. However, we identified five subfamilies of *NRAMP* genes in areca, suggesting that *AcNRAMPs* in areca may evolve a series of new functions.

The analysis of physicochemical properties helps decipher the potential functional natures of proteins. For instance, analysis of the protein pI value provides us with an important reference index for protein purification. Similar to other gene family members, 12 AcNRAMP proteins include both basic and acidic proteins [24,25,26]. All AcNRAMPs are considered thermostable proteins due to their Ai value being higher than 71. In contrast, most of AcNRAMP (10/12) are considered unstable proteins in a test tube due to their Ii values being higher than 40. In addition, except for AcNRAMP2, all AcNRAMP proteins have GRAVY values lower than zero, suggesting their soluble nature. Different organelle localization of NRAMP proteins shows different functions in plants. For example, a previous study shows that AtNRAMP6 is localized in the Golgi/trans-Golgi network and contributes to maintaining intracellular Fe homeostasis [27]. Additionally, AtNRAMP1 is localized on the plasma membrane and participates in Fe/Mn transportation [28]. In rice, most of the NRAMP protein members are also localized on the plasma membrane and are associated with the transport of various intracellular metal ions [17,29]. In the present study, subcellular localization predicts that most *AcNRAMP* genes are localized on the plasma membrane, indicating that most *AcNRAMP* genes transport ions on the membrane, and some *AcNRAMP* genes are located in chloroplast, indicating that they transport ions between organelles.

Numerous studies have found that plant *NRAMP* family genes are associated with the uptake and transport of various divalent metal ions [30]. In *A. thaliana*, *AtNRAMP1* expression can be induced under iron deficiency in the root system, and overexpression lines show high tolerance to iron. These results indicate that *AtNRAMP1* can regulate iron metabolism balance in roots [31]. In addition, *AtNRAMP1* also has the function of absorbing and transporting manganese and iron. Similar to *AtNRAMP1*, *AtNRAMP2* is mainly expressed in the root tip and is involved in manganese ion transport [32]. *AtNRAMP3* and *AtNRAMP4* expression is induced under iron deficiency stress and can regulate the homeostasis of iron and manganese ions in cells [33,34]. Furthermore, the *Atnramp6* mutant shows higher cadmium tolerance than the wild type [27]. Recently, in other plants, an increasing number of *NRAMP* genes have been proven to play a pivotal role in maintaining the balance of iron, manganese, and zinc in cells [35,36,37]. In our study, we also analyzed the expression profiles of *AcNRAMPs* in different tissues of areca. We found that *AcNRAMP1*, *AcNRAMP4*, *AcNRAMP6*, *AcNRAMP7,* and *AcNRAMP12* are up-regulated in leaves and veins, indicating that they may be involved in the transfer of metal elements. *AcNRAMP3*, *AcNRAMP4*, *AcNRAMP11,* and *AcNRAMP12* are up-regulated in the underground roots, indicating that these four genes may be involved in the accumulation of metal elements in the underground roots. Overall, the expression profile of *AcNRAMP* family genes varies between different organs, suggesting that these genes may exert their functions in specific areca tissues.

Studies have shown that seven *NRAMP* genes are present in *O. sativa*. Of these, *OsNRAMP1* is primarily located on the plasma membrane and is significantly up-regulated in response to Fe deficiency [38]. Aside from transporting Fe, *OsNRAMP1* also facilitates the movement of Cd across the membrane [39]. Moreover, *OsNRMAP5* plays a crucial role in the transportation of Fe, Mn, and Cd in *O. sativa*, thereby significantly contributing to the overall growth and development of the plant [40,41]. In this study, we further analyzed the expression profiles of *AcNRAMP* family genes in areca seedlings under the Zn/Fe deficiency stress. We observe that iron deficiency stress can induce the high expression of *AcNRAMP1*, *AcNRAMP9*, and *AcNRAMP10* in areca L1, suggesting that these genes might be the main iron transporter in areca during the iron deficiency stress. Meanwhile, the expression level of *AcNRAMP12* is higher in iron-deficient roots than in normal areca seedlings, indicating a vital role in iron uptake and transport in roots. Furthermore, the expression levels of *AcNRAMP1*, *AcNRAMP2*, *AcNRAMP3*, *AcNRAMP6*, *AcNRAMP8*, *AcNRAMP11*, and *AcNRAMP12* are significantly higher in the zinc-deficient group than those in the control group, suggesting that these genes may be involved in zinc ion transport. Additionally, we identified three *AcNRAMP* genes (*AcNRAMP2, AcNRAMP4*, and *AcNRAMP12*) that are closely related to areca roots’ tolerance to zinc deficiency stress.

## 4. Materials and Methods

### 4.1. Plant Material and Growth Conditions

An *A. catechu* cultivar “Reyan NO.1”, was selected as experimental material in this study. The six-month-old areca seedlings were cultured in Hoagland solution for adaptive cultivation. After two weeks, the seed bulbs of areca seedlings were removed from the base of the stem. Subsequently, the seedlings were cultured in whole nutrient solution (CK), Fe-deficient medium (CK without Fe-(Na)_2_EDTA), and Zn-deficient medium (CK without Zn^2+^), respectively.

### 4.2. Identification of AcNRAMP Genes in A. catechu

The conserved domain of NRAMP protein (PF01566) was obtained from the Pfam database. The AcNRAMP candidates were preliminarily retrieved from the *A. catechu* genome using hidden Markov model (HMM) search with an E-value lower than 10^−5^ based on the methods by Krogh et al. [42]. Pfam Database (http://pfam.xfam.org/) (accessed on 10 January 2023) and SMART (http://smart.embl-heidelberg.de/) (accessed on 10 January 2023) were used to determine the predicted protein as a member of the transporter gene family.

### 4.3. Analysis of Physicochemical Properties of AcNRAMPs

The physicochemical properties of AcNRAMP proteins were analyzed by using ExPASy6 (https://web.expasy.org/protparam/) (accessed on 12 January 2023) [43], and their subcellular localization was predicted using Plant-mPLoc (http://www.csbio.sjtu.edu.cn/bioinf/plant-multi/) (accessed on 12 January 2023) [44].

### 4.4. Analysis of Evolutional Relationships of NRAMP Genes in A. catechu, O. sativa, and A. thaliana

The sequences of AtNRAMPs and OsNRAMPs were obtained from TAIR (https://www.arabidopsis.org/) (accessed on 12 January 2023) and the Rice Annotation Project Database, (https://rapdb.dna.affrc.go.jp/download/irgsp1.html) (accessed on 12 January 2023), respectively. ClustalW was performed to align all sequences with default parameters, and then a phylogenetic tree was constructed using a neighbor-joining (NJ) method of MEGA-X [45]. The bootstrap replications were set as 1000 to test the NJ tree reliability. The Evolview8 program was employed to visualize the phylogenetic tree [46].

### 4.5. Analysis of Sequence Features, Chromosome Distribution, and Syntenic Relationships of AcNRAMPs

The MEME online (https://meme-suite.org/meme/tools/meme) (accessed on 12 January 2023) was performed to analyze conserved motifs shared among *AcNRAMP* genes [47,48]. The TBtools software (version. x64_1_0987657) was used to visualize the *AcNRAMP* gene structure according to the CDS and genomic DNA sequences of *AcNRAMP* genes [49]. Furthermore, the TBtools software was performed to visualize the information of genome-wide chromosomal density and the distribution of *AcNRAMPs* across all chromosomes of *A. catechu* based on the genome annotation files. The genes distributed on scaffolds were excluded. The TBtools were employed to analyze the syntenic relationships of *AcNRAMPs* genes in interspecies (*A. catechu* vs. *A. thaliana*, *A. catechu* vs. *C. nucifera*, and *A. catechu* vs. *O. sativa*) and intraspecies.

### 4.6. Analysis of Cis-Acting Elements

Upstream 2,000 bases from the first ATG of CDS of *AcNRAMP* genes were regarded as promoter sequences, which were acquired from the *A. catechu* genome. The cis-acting elements of these sequences were identified using PlantCARE (http://bioinformatics.psb.ugent.be/webtools/plantcare/html/) (accessed on 18 January 2023).

### 4.7. Transcriptomic Data Analysis of AcNRAMPs

In this experiment, three individual areca seedlings from each group were separately collected as three biological replicates. RNA from a total of 27 samples was extracted and subjected to high-throughput sequencing on the Illumina Hiseq 4000 platform at Biomarker Technologies Co. (Beijing, China). After filtering out low-quality reads, clean reads were aligned to the areca reference genome. Gene expression analysis was performed using BMKCloud (www.biocloud.net) (accessed on 18 January 2023). The expression profiles of *AcNRAMP*s under Fe and Zn deficiency conditions were analyzed by calculating the fragments per kilobase per million mapped reads (FPKM) of *AcNRAMP*s based on the RNA-seq data. The genes with expression levels that met the standards (|log2FC| > 1, FDR < 0.05, and *p*-value < 0.05) were considered differentially expressed genes (DEGs). The heatmap representing the expression levels of *AcNRAMP* genes was plotted using TBtools. The RNA-seq raw data were deposited at NCBI (accession number: PRJNA767949).

### 4.8. qRT-PCR Analysis

The RNA quick isolation kit (Tiangen, Beijing, China) was used to extract the high-quality total RNA from the areca samples, and the One-Step gDNA Removal and cDNA Synthesis SuperMix (Tiangen, Beijing, China) was used to synthesize cDNA. The qRT-PCR was conducted to analyze the relative expression of *AcNRAMPs*. Primer Premier 5.0 was used to design the qRT-PCR primers of the *AcNRAMP* genes (Table S1). The qRT-PCR used *β*-*actin* as an internal reference gene. The 20 μL qRT-PCR reaction mixture contained 10 μL of 2 × SYBR Green Master Mix (Tiangen, Beijing, China), 1 μL of forward and reverse primers, 1 μL of forward and reverse primers, 1 μL cDNA template, and 7 μL ddH_2_O. The qRT-PCR program follows 94 ℃ hold for 30 s, 40 cycles at 94 °C, hold for 30 s, and 60 °C hold for 30 s. The final results were analyzed by using the 2^−ΔΔCt^ method. All experiments were repeated with three biological and technical replicates.

## 5. Conclusions

NRAMP proteins play pivotal roles in plant biological processes, including the trafficking of metal ions, plant growth, and development. However, the information on *NRAMPs* in *A. catechu*, a commercially important medicinal plant of the palm family, is still unclear. In this study, the total members of the *NRAMP* gene family in the areca genome were identified. Subsequently, the sequence characteristics, gene structure, phylogeny, promoter sequence, and collinearity of all *AcNRAMP* genes were comprehensively analyzed. Furthermore, the expression levels of these genes in different tissues and under the condition of Fe and Zn deficiency were studied. The results reveal that *AcNRAMP* genes exert their functions in specific areca tissues and play key roles in areca response to Fe and Zn deficiency. This work provides a valuable reference for the in-depth study of the function of the *AcNRAMP* gene in coping with Fe and Zn deficiency stress in areca.

## Data Availability

The datasets presented in this study can be found in online repositories. The names of the repository/repositories and accession number(s) can be found in the article/supplementary material.

## Supplementary Materials

The supporting information can be downloaded at: https://www.mdpi.com/article/10.3390/ijms24087383/s1.
